# Ocular Manifestations in Patients Affected by p63-Associated Disorders: Ectrodactyly-Ectodermal Dysplasia-Clefting (EEC) and Ankyloblepharon-Ectodermal Defects-Cleft Lip Palate (AEC) Syndromes

**DOI:** 10.3390/jcm12237377

**Published:** 2023-11-28

**Authors:** Enzo Di Iorio, Filippo Bonelli, Raluca Bievel-Radulescu, Nicolò Decastello, Stefano Ferrari, Vanessa Barbaro, Diego Ponzin

**Affiliations:** 1 Clinical Genetics Unit, University Hospital of Padua, 35128 Padua, Italy; vincenzo.diiorio@unipd.it; 2 Department of Molecular Medicine, University of Padua, 35128 Padua, Italy; 3 Fondazione Banca Degli Occhi del Veneto, 30174 Venice, Italy; filippo.bonelli@fbov.it (F.B.); raluca.bievel@fbov.it (R.B.-R.); nicolo.decastello@fbov.it (N.D.); stefano.ferrari@fbov.it (S.F.); diego.ponzin@fbov.it (D.P.)

**Keywords:** p63, EEC (Ectrodactily Ectodermal Displasya-Clefting), syndrome, AEC (Ankyloblepharon-ectodermal defects-cleft lip/palate) syndrome, LSCD (limbal stem cell deficiency)

## Abstract

Background/Aims: The Ectrodactyly-Ectodermal dysplasia-Clefting (EEC) and Ankyloblepharon–ectodermal defect–cleft lip/palate (AEC) syndromes are rare autosomal dominant diseases caused by heterozygous mutations in the p63 gene. Patients are characterized by abnormalities of the skin, teeth, and hair and have limb defects, orofacial clefting and ectodermal dysplasia. In addition, they often show ocular surface alterations, leading to progressive corneal clouding and eventually blindness. Here, we present 8 cases describing patients affected by EEC (*n* = 6, with 5 sporadic and 1 familial cases) and AEC (*n* = 2, both sporadic cases) syndromes. We attempt to provide a description of the ocular disease progression over the years. Methods: Clinical examinations and monitoring of ocular parameters for the assessment of limbal stem cell deficiency were constantly performed on patients between 2009 and 2023. Quantitative data and comparison with existing cases described in the literature are reported. Results: The therapies supplied to patients were essential for the management of the symptoms, but unfortunately did not halt the progression of the pathology. Conclusions: A constant monitoring of the patients would help avoid the sudden worsening of symptoms. If the progression of the disease slows down, it would allow for the development of newer therapeutic strategies aimed at correcting the genetic defect.

## 1. Introduction

The proper functionality of p63, a member of the p53 transcription factor family, is essential for the correct development of ectodermal tissues. Two different promoters originate two isoforms, one containing a transactivation domain, TAp63, and another one without it, ΔNp63 [1]. Further splicing activity produces three different isoforms: α, β, and γ [1,2]. The refined interplay between the different isoforms is responsible for the proper development of the ectodermal tissues, particularly the epithelia [3,4,5]. After birth, p63 expression is maintained in the basal epithelia by stem-like cell populations [6]. Among them, the corneal epithelium mostly relies on ΔNp63α activity to maintain the proliferative potential of limbal epithelial stem cells (LESCs), which are crucial for the regenerative potential of the ocular surface [7]. Several works have reported that embryos of double KO mutants for p63 have no epithelia and die before birth due to dehydration [8,9,10]. Heterozygous mutations in p63 give rise to a plethora of rare syndromes that are very similar to each other [11]: Ectrodactyly-Ectodermal dysplasia Clefting (EEC) syndrome, Rapp Hodgkin (RHS) syndrome, Hay Wells or Ankyloblepharon-Ectodermal defects-Cleft lip/palate (AEC) syndrome, limb-mammary (LMS) syndrome, AcroDermato-Ungual-Lacrimal-Tooth (ADULT) syndrome, and Split-Hand/Foot Malformation type 4 (SHFM4) syndrome.

**Figure 1:**
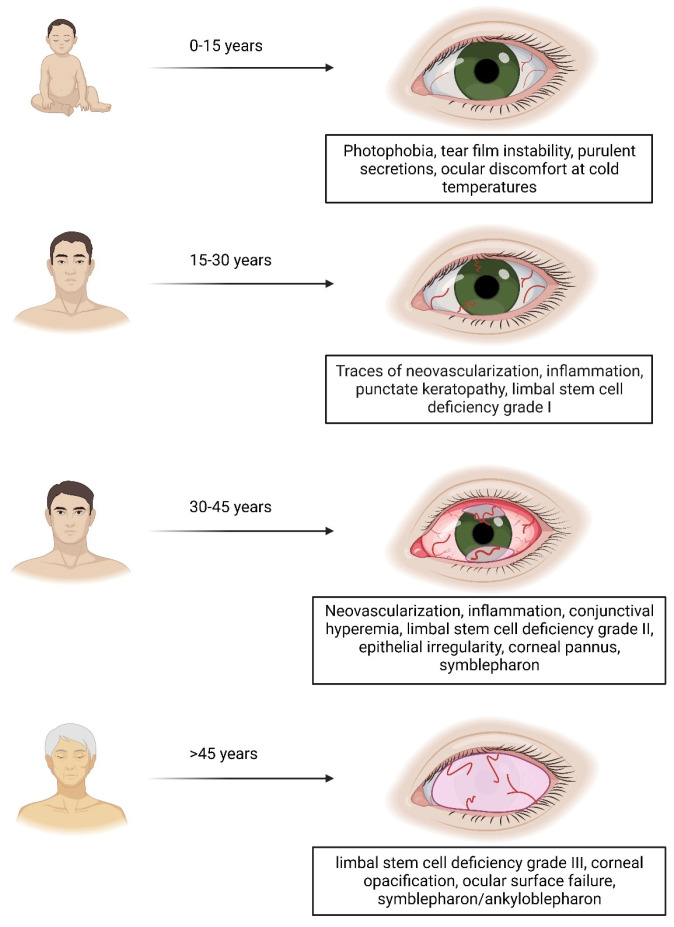
A graphical representation of ocular manifestations linked to progressive limbal stem cell deficiency (LSCD), that ultimately results in vision neovascularization, loss. Created with BioRender.com, accessed on 5 November 2023.

Among them, the EEC and AEC syndromes are characterized by ectodermal defects such as split-hand/split-foot malformation, epidermal, hair and nail abnormalities, labiopalatoschisis, and tooth disorders (particularly in number and eruption timing) [12,13]. Moreover, several patients report ocular manifestations linked to progressive limbal stem cell deficiency (LSCD), which ultimately results in vision loss [14] (Figure 1). Of particular relevance, mutations involving Arginines in positions 279 and 304 have been shown to cause the most detrimental effects for the eye, with R279H and R304Q representing the worst case scenario [15].

In this paper we report a series of 8 cases with ocular manifestations due to EEC or AEC syndromes. The age of the patients ranges from 6 to 69 years, thus covering a broad spectrum of how the pathology develops and the ocular alterations progress along the years.

## 2. Materials and Methods

The patients (*n* = 8) provided their written informed consent to participate in this study after approval from the Ethical Committee for Clinical Trials of Padua, Italy (Prot. 4503/AO/18). Written informed consent was obtained from the individuals for the publication of any potentially identifiable images or data included in this article.

Clinical examinations were performed between 2009 and 2023 at Fondazione Banca degli Occhi del Veneto (Venice, Italy).

The clinical examinations performed on patients during the routine follow-up visits and the methods and instruments used to carry out the analyses are reported in Table 1.

As for the genetic analyses, sequence variants were named according to the guidelines published by the Human Genome Variation Society (http://www.hgvs.org/mutnomen/, accessed on 5 November 2023). The position of mutation is given according to the original published TA-TP63a sequence (GenBank accession no. AF075430), which does not include the 39 additional aminoacids at the N-terminus as reported by GenBank (accession no. AF091627; gi:3695081).

The ocular parameters that were monitored for the assessment of limbal stem cell deficiency in EEC/AEC patients are summarized in Table 2.

## 3. Results

### 3.1. Case 1

**Figure 2:**
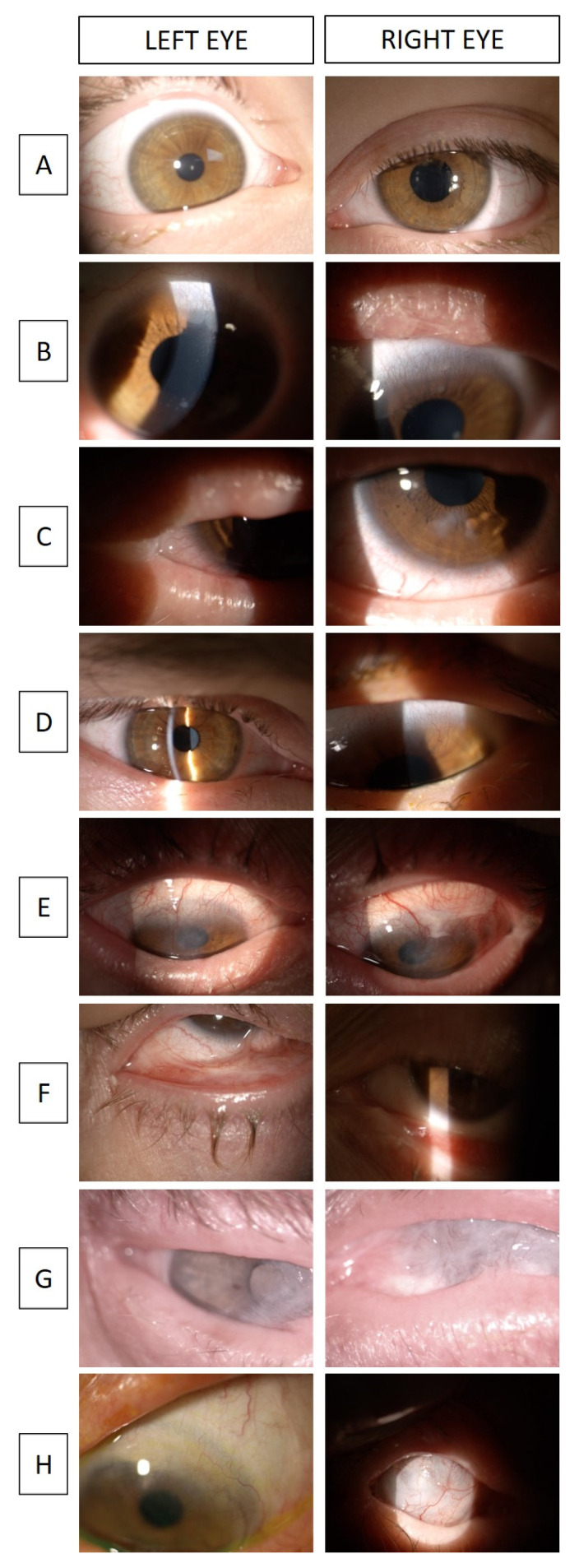
Ocular characteristics of patients. (**A**) A patient of pediatric age, affected by EEC syndrome. No signs of corneal pannus were observed, and the corneas are both transparent. No Meibomian gland orifices are present; (**B**) a patient ranging between 10 and 20 years of age, affected by AEC syndrome. The palisades of Vogt are absent in both eyes. The corneal epithelium is still intact; (**C**) a patient ranging between 10 and 20 years of age, affected by AEC syndrome. The palisades of Vogt are absent in both eyes; (**D**) a patient in their early 30 s, affected by EEC syndrome. A mild epitheliopathy is present in both eyes. Signs of corneal punctate epitheliopathy and neovascularization are observed in the left eye; (**E**) a patient in their early 40 s, affected by EEC syndrome. Corneal pannus and neovascularization in the superior sector of both corneas are present, as well as corneal epitheliopathy. In the right eye, a corneal pannus covered the supero-temporal area, with vessels invading the cornea; (**F**) a patient in their early 40 s, affected by EEC syndrome. Pannus was observed in both eyes. Symblefaron is also present in the right eye, as well as sub-epithelial fibrosis of the conjunctiva; (**G**) a patient in their early 60s, affected by EEC syndrome. Complete conjunctivalization in both eyes is present, with bilateral symblefaron and total impairment of the ocular surface; (**H**) a patient ranging between 60 and 70 years of age, affected by EEC syndrome. Simblefaron/anchiloblefaron and squamous metaplasia were present in the right eye, while an initial pannus was observed in the inferior/nasal segment of the left eye. The palisades of Vogt are absent. Abbreviations: EEC, Ectrodactyly-Ectodermal Dysplasia-Clefting.

A 6-year-old girl born in Italy, with unrelated and unaffected parents, was diagnosed with EEC syndrome at birth (Table 3). The patient is the youngest of two siblings and has an unaffected sister. The molecular analysis, performed at the age of 5, revealed the presence of the genomic variant 953G>A (p. Arg318His) in exon 7 of TP63, which confirms the R279H mutation in the DNA binding domain (DBD). The patient underwent surgical labiopalatoschisis and bifid uvula correction at the age of 6 months. At the same time, excision of a digitiform appendage in the first interdigital space of the left foot was carried out. Surgical correction of the left cleft hand and separation of the first and second digits of the right foot were performed. Ocular manifestations started at the age of 3 in the form of bilateral secretion of purulent liquid following pressure on the eye. The patient was then recommended to undergo flushing of the tear ducts. We examined her for the first time at the age of 5. Whilst stable, OCT analysis revealed a thin cornea in both eyes, with the left cornea being 425 µm thick and the right one being 437 µm thick. Meibomian glands were almost absent and tear Break-Up Time (TBUT) revealed tear film instability, with values of 8 and 7 s being reported for the left and right eyes, respectively. The palisades of Vogt were detected in both eyes. No signs of corneal pannus were observed at inspection and the cornea was transparent (Figure 2A). The patient reported discomfort during the coldest months of the year, but no inflammatory infiltrates were observed at inspection. The patient was strongly photophobic. Palpebral hygiene, once/twice per day, was recommended.

### 3.2. Case 2

A 17-year-old girl, born in Italy, with unrelated and unaffected parents. She is the younger sister of case 3 (Table 3). The molecular analysis revealed the presence of the genomic variant 1568T>C (p. Leu523Pro) in exon 13 of TP63, which confirms the L523P mutation in the sterile alpha motif (SAM) domain, a novel missense mutation that is known to cause the AEC syndrome [22]. The patient was born with labiopalatoschisis, facial dysmorphism, feet malformations, and collodion baby. The first exam we performed was when the patient was 3 years old, with the parents reporting allergic reactions involving the ocular surface and photophobia. At inspection, the palisades of Vogt were already absent, as well as the meibomian glands. Conjunctival chemosis, tear film instability, and scarce tear meniscus were detected during the examination (Figure 2B). The progression of the ocular manifestations was monitored once a year, and the situation was stable at the last visit in March 2023. The patient was only observed to have some mucus secretions in the eyes, and some ciliae in dystichiasis were removed. The patient uses daily ocular lubricant, anti-inflammatory eyedrops, and lipidic-based tear film stabilizers.

### 3.3. Case 3

A 20-year-old girl, born in Italy, with unrelated and unaffected parents. She is the older sister of case 2 (Table 3). The molecular analysis, performed at the age of 3, revealed the presence of the genomic variant 1568T>C (p. Leu523Pro) in exon 13 of TP63, which confirms the L523P mutation in the SAM domain, which is known to cause AEC syndrome. The patient was born with palatoschisis, facial dysmorphism, feet malformations, diffused dermatosis, and nail dystrophies. At birth, the patient presented low levels of B and T helper lymphocytes and after one year she was hospitalized for interstitial bronchopneumopathy, which was accompanied by diarrhea, vomit, anuria, and strong dehydration. We first examined the patients at the age of 6 when she was brought in for a routine eye sight visit. The parents reported that the child suffered from photophobia and seasonal ocular allergies. When examined, a small corneal leucoma was identified in the right eye, while the left eye was healthy. The palisades of Vogt were already absent in both eyes. Analysis of adnexa revealed the absence of Meibomian glands, which resulted in unstable tear film, scarce meniscus, and mucus secretions. Schirmer’s test was performed with a result of 8 mm for both eyes. The patient remained stable for three years, even if maintaining the corneal leucoma and presenting madarosis. At the age of 10, mild epithelial irregularities were detected in both eyes, with traces of conjunctival chemosis. At the age of 12, the patient suffered from corneal abrasion in the lower area of right eye and was treated with antimicrobials (Exocin eye drops), but a further progression in corneal epithelial irregularities was observed during routinely control. The patient was stable when last visited in March 2023 (Figure 2C) and was suggested to use daily ocular lubricant, anti-inflammatory eyedrops, and lipidic-based tear film stabilizers.

### 3.4. Case 4

A 31-year-old male, born in Italy, with unrelated and unaffected parents was diagnosed with EEC syndrome at birth (Table 3). Further genetic investigation on peripheric blood, carried out at the age of 18, revealed that the patient was heterozygous for the genomic variant 953G>A (p. Arg279His) in exon 7 of TP63, confirming to the R279H mutation in the DBD. The patient was born with labiopalatoschisis, cleft hands and feet, but has no history of other disorders. At the age of 19, he contacted our center complaining about periodic bulbar hyperemia, ocular surface inflammation, palpebral oedema and oily, filamentous tearing. Inspection revealed bilateral mild punctate keratopathy in the lower area of the eye. At the age of 28 he was diagnosed with the first signs of corneal neovascularization and the palisades of Vogt were almost absent.

During the last visit, in December 2022, a mild epitheliopathy in the lower sector of the right eye was diagnosed, with traces of hyperemia and neovascularizazion. In the left eye, the patient was suffering from punctate keratopathy, as determined following fluorescein staining, with signs of hyperemia and neovascularization. Corneal thickness was thin in both eyes, with the right eye being 471 µm thick and the left eye being 439 µm thick. Corneal epithelium had minimal and maximal values of thickness of 43 and 50 µm in the right eye and 33 and 57 µm in the left eye, respectively. The palisades of Vogt were absent in both eyes. Inflammation together with purulent secretions and ocular burning were present in both eyes. Meibomian glands were missing and tearing was sebaceous and filamentous (Figure 2D). The patient reports strong photophobia. Lacrimal substitutes and ocular lubricant have been prescribed.

### 3.5. Case 5

A 42-year-old male with maternal familiarity was diagnosed with EEC at birth (Table 3). Further genetic investigation, at the age of 28, highlighted the genomic alteration 953G>A (p. Arg279His) in exon 7 of TP63, causative for the R279H mutation in the DBD. The patient was born with ectrodactily in all limbs, but with no labiopalatoschisis.

As for the ocular manifestations, the patient was stable until the age of 30, with no corneal epithelial defects. However, tear film instability, Meibomian gland obstruction and partial aplasia, absence of Palisades of Vogt and conjunctival hyperemia were detected when visited. At the age of 32, fluorescein staining of the ocular surface revealed bilateral punctate keratopathy. One year later, when examined, corneal pannus in the superior area of both eyes was observed, with corresponding epithelial irregularities. At the last visit, in March 2022, the patient suffered from corneal pannus and neovascularization in both eyes, but mostly in the right eye. Corneal epithelial defects were localized where the pannus was present. In the right eye, the pannus covered the supero-temporal area of the eye, with a vessel invading the cornea. Mild sclerosis of the lens was detected. In the left eye, the pannus had invaded the cornea from the supero-temporal and inferior regions, with neovascularization progressing from both sides of the eye. Both eyes presented punctate keratopathy when stained with fluorescein. OCT analysis revealed that both the right and left corneas were thin, with values of 308 µm and 333 µm, respectively. Similarly, corneal epithelial thickness had values of 33 and 75 µm in the right eye and 24 and 55 µm in the left eye (minimum and maximum values, respectively). The palisades of Vogt were absent in both eyes. Analysis of the ocular adnexa revealed Meibomian gland aplasia, with existing ducts being obstructed. Consequently, strong tear film instability was detected, with a TBUT value of 3 s. Schirmer’s test gave a value of 25 mm. In both eyes a marked hyperemia was observed, together with chronic inflammation of the ocular surface (Figure 2E). The patient reported photophobia, ocular fatigue, and has trouble when driving during night hours. The patient is currently under daily steroid eye drops, tear substitutes, and wears scleral lenses.

### 3.6. Case 6

A 44-year-old female, born in Italy, with unrelated and unaffected parents, was diagnosed with EEC syndrome at birth (Table 3). Further genetic analysis revealed the 952C>T (p. Arg279Cys) genomic alteration in intron 7 of TP63, responsible for the R279C mutation in the DBD.

Significant ocular manifestations began at the age of 29 and slowly evolved over time. Due to a severe tear film dysfunction, the patient was implanted with tear plugs in the right eye, while palpebral reflexes did not allow for the procedure to be carried out in the left eye. One month later, during routine examination, inspection revealed positivity at fluorescein staining in the left eye, which correlated with a TBUT of 2 s. Due to this strong tear film instability, the patient was recommended to undergo tear duct flushing. Further controls at the age of 31 showed large, central, and superior leucoma in both eyes, spreading more in the right than in the left eye. Ocular pannus was observed in the superior segment of both eyes, and more developed in the right eye. No signs of keratopathy were observed in both eyes and neovascularization was stable. From the age of 32, symblepharon was observed in the lower-nasal area of the left eye, and at the age of 40 symblepharon also appeared in the lower segment of the right eye. During the last visit, in March 2022, corneal pachymetry showed corneal thickness values of 403 µm in right eye and 415 µm in left eye, with minimal epithelial thickness of 30 µm and 24 µm (in the right and left eyes, respectively). Lens sclerosis was detected in the right eye. Ocular adnexa inspection confirmed meibomian gland aplasia, with consequent tear film instability and scarce tear meniscus (Figure 2F). In the right eye, some eyelashes in trichiasis were observed. The patient is under daily tear substitutes and steroid eye drops.

### 3.7. Case 7

A 61-year-old female, mother of case 5, with no familiarity, was diagnosed with EEC syndrome at the age of 29, following an initial diagnosis of cicatritial pemphigoid (Table 3). Results from molecular analysis on peripheral blood reported the presence of the genomic alteration 953G>A (p. Arg279His) in exon 7 of TP63, causative for the R279H mutation in the DBD.

The patient was born at the seventh month of pregnancy. No ectrodactily or labiopalatoschisis were present at birth, however hexadactyly in the left hand was surgically corrected in the first year of life. Ectodermal dysplasia manifested with dry and hypopigmented skin, nail alterations, and hair hypopigmentation. Strong anomalies in tooth eruption, particularly in number and timing, were detected. During pregnancy, alterations in genitourinary mucosae were observed, concomitant with a human papillomavirus (HPV) infection.

Ocular manifestations started at the age of 24, with initial signs of visual decay. The patient also reported recidivism in blepharitis and conjunctivitis. From the age of 26, she started experiencing strong vision loss in the left eye due to corneal pannus advancement. At the age of 48, the patient was diagnosed with complete conjunctivalization in the left eye, and a large leucoma with calcification in the lower hemisphere of the right eye. At the last visit, in March 2018, bilateral symblepharon of internal cantus was detected. The palisades of Vogt were completely absent. Meibomian gland aplasia was reported, with instable tear film (Figure 2G). The patient reported scarce visus and strong photophobia. In the following years the patient experienced lens opacification and underwent cataract surgery in the right eye, with bilateral total impairment of the ocular surface and severe dyslacrimia.

### 3.8. Case 8

A 69-year-old male, with no familiar history of EEC syndrome, was diagnosed at the age of 59, following an initial diagnosis of ocular pemphigoid (Table 3). Genetic investigation revealed two different genomic variants in the TP63 gene, namely c.952 C>T (p. Arg318Cys) and 1135C>G (p. Arg379Gly), which give rise to the mutations R279C and R340G, respectively. The patient presents with ectrodactyly in both hands and feet, and was born with palatoschisis. At the age of 62, due to infections following cataract surgery on the right eye, the patient underwent two successive corneal transplants and eventually amniotic membrane graft. At the last visit, in March 2023, simblefaron/anchiloblefaron, surface squamous metaplasia and fornix secretions were present in the right eye while an initial ocular pannus was observed in the inferior/nasal segment of the left eye, as well as a subconjunctival fibrosis. Both the palisades of Vogt and the meibomian glands were absent (Figure 2H).

## 4. Discussion

The Ectrodactyly-Ectodermal dysplasia Clefting syndrome (MIM#604292) affects about 1–9/100,000 newborns (according to www.orpha.net, accessed on 30 May 2023) [14,23]. Heterozygous missense mutations result in haploinsufficiency, as about 50% of the proteins can complex in the tetrameric structure and carry out their biological function [5]. While non-life threatening, EEC causes multiple functional impairments in affected people. Limb deformities, labiopalatoschisis, and skin/hair abnormalities are the most evident features of the disease. However, the most invalidating problem is the progressive limbal stem cell deficiency affecting most of the patients [12,13,14], which ultimately results in gradual vision loss and blindness. It was previously believed that the ocular manifestations in EEC syndrome were linked to Meibomian and lacrimal gland dysfunction, tear film instability, and therefore to the associated dry eye disease. EEC syndrome is, in fact, associated with Meibomian gland agenesis, with produces severe evaporative dyslacrimia and desiccation of the ocular surface epithelium. Whilst relevant for the progression of the disease, we previously demonstrated that LSCD is the main cause for corneal thinning, neovascularization, corneal pannus, and lastly conjunctival epithelial ingrowth [14]. It has been widely demonstrated that p63 is expressed in the basal layer of adult epithelia, and plays a key role in maintaining stemness and proliferative activity [6]. The corneal epithelium is constantly exposed to environmental insults, and hence requires a constant and permanent renewal [24,25]. Therefore, heterozygous mutations in p63, combined with the continuous proliferative stress, leads to an early exhaustion of the limbal stem cell pool.

The Ankyloblepharon–ectodermal defect–cleft lip/palate (AEC) syndrome (MIM#106260), also known as Hay–Wells syndrome, shares many features that are also common to the EEC syndrome, being characterized by the presence of ankylobleopharon (fibrous strands of tissue that can partially or completely fuse the upper and lower eyelids) and ectodermal abnormalities (including sparse and frizzy hair, skin erosion, nail alterations, dental changes, and hypohidrosis), associated with a clefting of the lip and/or the palate. Affected individuals may also have narrowing (atresia) or absence of the opening in the edge of each eyelid that is linked to the tear duct (lacrimal punctata). This can lead to obstruction of the tear ducts and predispose to recurrent eye crusting and conjunctivitis. Many individuals with AEC report chronic dry eyes. Chronic inflammation of the eyelids (blepharitis) has also been reported [26].

In this work, we report six cases of EEC and two of AEC syndromes with ocular complications that we currently follow in our institution. As the youngest patient was 6 years old and the oldest patient was 69 years old, we have had the chance to observe the progression of the disease along the years.

Our youngest patient, aged 6, shows the typical features of early-stage EEC syndrome. The most relevant pathological sign is, for instance, Meibomian and lacrimal gland dysfunction, which results in tear film instability and posterior blepharitis. However, Palisades of Vogt are present, and the corneal epithelium, despite thin, is still intact. Symptoms observed in this patient are coherent with other EEC cases of similar age, especially for what concerns ocular adnexa issues [27,28,29]. However, interestingly, the patient does not suffer from epiphora, which was common in other patients and solved through dacryocystorhinostomy.

The two sisters affected by AEC syndrome, who were previously studied by our group for their novel mutation and for the germline mosaicism that caused such a singular case [22], present again a mild phenotype, which is compatible with their young age. Meibomian gland dysfunction, tear film instability and conjunctival chemosis were the common traits in both girls, with the older one presenting some alterations in corneal epithelium. Other papers reported the cases of young patients with ages similar to cases 2 and 3, but in all of them more deteriorated clinical conditions were described. [27,28,29,30]. As far as we know, only one other case of siblings from non-affected parents has been reported so far, but the mutations are not known [31].

It is difficult to compare our case 4 to others reported in the literature, since very few people of the same age have been reported. A recent work described a man aged 31 with a more severe phenotype, including complete dysfunction of the Meibomian glands, conjunctival fibrosis, and corneal neovascularization [32]. Of note, the time between the third and the fifth decade of life represents the most interesting for the progress of the disease, as the ocular features undergo a rapid decay. By contrast, case 4 shows a stable situation, with only some initial corneal neovascularization. This might be due to the fact that case 4 is not characterized by a complete loss of function of the Meibomian glands.

Family histories similar to those described in cases 5 and 7 are reported in the literature [29,33,34,35,36,37]. McNab and colleagues [29] described the case of a mother with mild EEC syndrome (no cleft hand/foot, only polydactyly in left hand, and syndactyly in her feet) giving birth to a progeny of four siblings, with two affected by EEC syndrome and having both labiopalatoschisis and ectrodactyly. This closely resembles our findings: a mother (case 7) having a mild phenotype (no cleft hand or foot, but ocular failure), while the son (case 5) had ectrodactyly in the four limbs and worsening ocular manifestations.

Case 6 is similar to case 5, displaying the first signs of ocular degeneration, with an initial pannus and neovascularization.

Case 8 is a unique case of a patient carrying two pathogenetic mutations, R279C and R340G, in two different exons of the p63 gene (exons 7 and 9, respectively), and with the last one being novel and classified as likely pathogenetic. Despite that, the ocular manifestations are not among the most severe so far observed. A likely explanation might be that the R340G mutation does not fall in the DBD of p63, where all the mutations causing the EEC syndrome are localized. Similarly, the R279C mutation is localized in a well conserved sequence between the DBD and the tetramerization domain (TD), and it is likely not correlated with ocular defect phenotypes. A somatic mosaicism might be guessed, with some cells carrying the R279C and others carrying the R340G mutations, respectively. This will have to be assessed by a thorough sequencing analysis.

## 5. Conclusions

The management of the corneal defects associated with EEC and AEC syndromes is challenging and, to date, no definitive cures are available.

The patients involved in this investigation underwent a treatment plan that relied exclusively on steroids, intentionally omitting any anti-inflammatory drugs. The steroid treatment was adjusted in concentration in accordance with their respective clinical needs.

All subjects, as part of the prescribed treatment, diligently maintained eyelid cleanliness and applied warm compresses to their eyes every night. An integral aspect of the treatment protocol involved the daily administration of eye drops containing hyaluronic acid, in order to augment the overall therapeutic strategy by fostering ocular hydration and optimizing the ocular surface environment. The regular application of hyaluronic acid was deemed essential for its potential benefits in maintaining ocular lubrication and supporting the healing process.

What have we learnt in the last 10 years, and to what extent have the therapies supplied to patients altered the progression of the disease? The therapeutic strategy outlined above was crucial and is currently still pivotal for the management of the symptoms, but unfortunately has not halted the progression of the pathology. The ocular defects of our patients with EEC or AEC syndromes still remain invalidating and become chronic conditions, for which we feel no definitive treatments are currently available. However, while the latter is true, clinical studies for testing new drugs or cell/gene-based therapies can hardly be undertaken as the number of patients is limited. In addition, a large clinical variability is seen among patients, even in the presence of the same mutation in the p63 gene, thus suggesting that multiple factors (of genetic and environmental origin) are likely to interfere and overlap.

Therefore, with such overwhelming challenges, what type of cures or care can clinicians offer to these patients? Surely, a constant monitoring of the patients would help avoiding the sudden worsening of symptoms. Prospectively, this might also have further advantages: if the progression of the disease slows down, it would allow the development (and hopefully, application) of newer therapeutic strategies aimed at correcting the genetic defect [15,38].

In addition, a closer collaboration between clinicians and scientists with patients and patients’ association would be desirable and much awaited. A major gap to fill would be the development of guidelines for the treatment of the ocular morbidities in patients with EEC and AEC syndromes, similarly to what expert groups and patient associations (Aniridia Europe) have recently done for Aniridia, by defining the “Dos and Donts” for patient care (https://www.aniridia.eu, accessed on 5 November 2023—Clinical guidelines). If established, such synergy should allow for the development of a more optimal therapeutic strategy, starting from when the molecular diagnosis is made and monitoring the patients throughout their lives at well-defined time points. Last, but not least, patients’ compliance with the therapeutic strategy set by clinicians should be considered both crucial and essential, in order not to invalidate the outcome of the adopted treatments.

## Figures and Tables

**Table 1:** Clinical examinations performed to assess the pathological progression of the ocular surface disease in EEC/AEC patients.

Clinical Examination	Analysis Method
Anterior segment	Slit lamp (CSO SL9900 LED c/PC)
Epithelial transparency	
Epithelial defects	
Corneal neovascularization	
Corneal opacity	
Inflammation	
Presence of palisades of Vogt	
Presence of Meibomian glands	
Fundus	
Corneal pannus extension	
Visual acuity	Snellen test
Intraocular pressure (IOP)	ICare tonometer
Epithelial integrity	Slit lamp + Fluorescein staining (Grading Oxford)
Ocular dryness	Schirmer’s test Tear Break-Up time (TBUT) test
Corneal thickness	Anterior Segment Optical Coherence Tomography (AS-OCT CSO MS39)

**Table 2:** Ocular parameters monitored for the assessment of limbal stem cell deficiency (LSCD) in EEC/AEC patients.

Ocular Parameter	Clinical Significance	Assessment Method	Grading Scale
Epithelial Transparency	Decreased transparency may indicate limbal stem cell deficiency	Slit lamp examination (cobalt blue filter) with fluorescein dye	Grade 0: Normal, clear corneal epithelium. Grade 1: Mild epithelial haze with minimal symptoms and no or mild corneal staining. Grade 2: Moderate epithelial haze with moderate symptoms and mild-to-moderate corneal staining. Grade 3: Severe epithelial haze with severe symptoms and significant corneal staining or vision loss [16].
Recurrent epithelial defects	Corneal epithelial defects can indicate limbal stem cell deficiency	Slit lamp examination (cobalt blue filter) with fluorescein dye	Grade 0: No recurrent epithelial defects. Grade 1: Superficial punctate keratitis or minor epithelial defects with minimal symptoms and no or mild corneal staining. Grade 2: Moderate recurrent epithelial defects with moderate symptoms and mild-to-moderate corneal staining. Grade 3: Severe recurrent epithelial defects with severe symptoms and significant corneal staining or erosion [16].
Corneal neovascularization	Limbal stem cell deficiency can lead to corneal neovascularization	Slit lamp examination with white light	Grade 0: No neovascularization. Grade 1: Superficial neovascularization limited to the peripheral cornea (≤2 mm from the limbus). Grade 2: Superficial neovascularization extending beyond the peripheral cornea (>2 mm from the limbus) or deep stromal neovascularization limited to the peripheral cornea. Grade 3: Superficial neovascularization extending to the central cornea or deep stromal neovascularization extending beyond the peripheral cornea. Grade 4: Total corneal neovascularization, with or without associated corneal opacity [17].
Corneal opacity	Limbal stem cell deficiency can lead to corneal opacification	Slit lamp examination with white light	McDonald–Shadduck grading system: This grading system is based on the amount of corneal surface area covered by the opacity and ranges from 0 to 4. The grading is as follows: Grade 0: No corneal opacity. Grade 1: Less than 25% of the cornea covered by opacity. Grade 2: 25–50% of the cornea covered by opacity. Grade 3: 50–75% of the cornea covered by opacity. Grade 4: More than 75% of the cornea covered by opacity [18].
Inflammation	Inflammation can indicate limbal stem cell deficiency	Slit lamp examination with white light	This parameter can be evaluated using a scale from 0 to 4, with 0 being no inflammation and 4 being severe inflammation.
Bulbar hyperemia	Increased blood flow to the eye can indicate limbal stem cell deficiency	Slit lamp examination with white light	Efron Grading Scale Grade 0: No hyperemia. Grade 1: Slight hyperemia with some injection visible. Grade 2: Mild hyperemia with injection easily visible. Grade 3: Moderate hyperemia with pronounced injection. Grade 4: Severe hyperemia with very pronounced injection [19].
Palpebral rim abnormalities	Abnormalities in the eyelid can indicate limbal stem cell deficiency	External examination	Grading for palpebral rim abnormalities based on the severity of the abnormality and the extent of the lid involvement: Grade 0: Normal palpebral rim with no abnormalities. Grade 1: Mild palpebral rim abnormalities, involving less than half of the lid margin, including minimal notching, mild entropion, and minor lash abnormalities. Grade 2: Moderate palpebral rim abnormalities, involving more than half of the lid margin; including moderate notching, moderate entropion, and moderate lash abnormalities. Grade 3: Severe palpebral rim abnormalities, involving the entire lid margin; including severe notching, severe entropion, and severe lash abnormalities [20].
Corneal pannus	Limbal stem cell deficiency can lead to corneal pannus formation	Slit lamp examination with white light	Grading system for corneal pannus based on its location and extent: Grade 0: No pannus Grade 1: Peripheral pannus with 20–30% cornea affected. Grade 2: Mid- peripheral pannus with 30–50% cornea affected. Grade 3: Mid- peripheral pannus more than 50–70% cornea affected. Grade 4: Central pannus, more than 70–90% cornea affected [21].

**Table 3:** Summary of patient characteristics. The above table reports patient information, including age, gender and mutation, and clinical features, such as presence of palisades of Vogt and Meibomian glands. Epithelial transparency, recurrent epithelial defects, corneal neovascularization, corneal opacity, inflammation, bulbar hyperemia, palpebral rim abnormalities, and corneal pannus were scored according to the grading systems provided in Table 2 of the Materials and Methods section.

Case n	Age	Mutation	Eye	Palisades of Vogt	Meibomian/Lacrimal Glands	Epithelial Transparency [16]	Recurrent Epithelial Defects [16]	Corneal Neovascularization [17]	Corneal Opacity [18]	Inflammation [19]	Bulbar Hyperemia [20]	Palpebral Rim Abnormalities [21]	Corneal Pannus [22]
1	6	R279H	RE	+	Partial aplasia	0	0	0	0	1	1	1	0
			LE	+	Partial aplasia	0	0	0	0	1	1	1	0
2	17	L523P	RE	-	Absent	1	1	2	1	2	2	3	1
			LE	-	Absent	1	1	2	1	2	2	3	1
3	20	L523P	RE	-	Absent	2	2	2	2	2	3	3	2
			LE	-	Absent	2	2	2	2	2	3	3	2
4	31	R279H	RE	-	Absent	1	1	1	1	1	1	1	0
			LE	-	Absent	1	1	2	1	1	1	1	1
5	42	R279H	RE	-	Partial aplasia	2	2	3	3	3	4	1	3
			LE	-	Partial aplasia	2	2	3	3	3	4	1	3
6	44	R279C	RE	-	Absent	2	2	3	2	1	1	2	2
			LE	-	Absent	2	2	2	1	1	1	2	1
7	61	R279H	RE	-	Absent	3	3	4	4	1	1	3	4
			LE	-	Absent	3	3	4	4	1	1	3	4
8	69	R279C; R340G	RE *	-	Absent	3	3	4	4	1	3	2	4
			LE	-	Absent	2	2	1	1	1	1	2	1

Abbreviations: LE, lefte eye; RE, right eye. * patient underwent ocular surface failure following repeated surgical interventions.

## Data Availability

The data that support the findings of this study are available from the corresponding author upon reasonable request. Patients or the public were not involved in the design, or conduct, or reporting, or dissemination plans of our research.
